# The efficacy and safety of combined therapy with endobronchial tamponade and bronchial artery embolization for massive hemoptysis

**DOI:** 10.1186/s12890-024-03116-4

**Published:** 2024-07-03

**Authors:** Chaohui Lin, Yanfeng Chen, Donglu Cai, Zhiyu Chen, Zhuli Peng, Huiting Lai, Dexin Liu

**Affiliations:** Department of Radiology, The Second Affiliated Hospital of Fujian Medical University, No. 950 Donghai Street, Fengze District, Quanzhou, Fujian Province 362000 China

**Keywords:** Massive hemoptysis, Bronchial artery embolization, Endobronchial tamponade, Efficacy

## Abstract

**Background:**

Massive hemoptysis is characterized by its life-threatening nature, potentially leading to airway obstruction and asphyxia. The objective of this study was to evaluate the clinical effectiveness of combining endobronchial tamponade with bronchial artery embolization (BAE) in the treatment of massive hemoptysis.

**Methods:**

Between March 2018 and March 2022, a total of 67 patients with massive hemoptysis who underwent BAE were divided into two groups: the combination group (*n* = 26) and the BAE group (*n* = 41). Technical and clinical success rates were assessed, and adverse events were monitored following the treatment. Blood gas analysis and coagulation function indicators were collected before and after the treatment, and recurrence and survival rates were recorded during the follow-up period.

**Results:**

All patients achieved technical success. There were no significant differences in the clinical success rate, recurrence rates at 3 and 6 months, and mortality rates at 3 months, 6 months, and 1 year between the combination group and the BAE group. However, the hemoptysis recurrence rate at 1 year was significantly lower in the combination group compared to the BAE group (15.4% vs. 39.0%, *P* = 0.039). No serious adverse events were reported in either group. After treatment, the combination group showed higher levels of arterial partial pressure of oxygen (PaO2), oxygenation index (PaO2/FiO2), fibrinogen (FIB), and D-dimer (D-D) compared to the BAE group (*P* < 0.05). Multivariate regression analysis demonstrated a significant correlation between combined therapy and hemoptysis-free survival.

**Conclusion:**

Combination therapy, compared to embolization alone, exhibits superior efficacy in improving respiratory function, correcting hypoxia, stopping bleeding, and preventing recurrence. It is considered an effective and safe treatment for massive hemoptysis.

## Background

Massive hemoptysis is characterized by its life-threatening nature, potentially leading to airway obstruction and asphyxia [1]. In the literature, massive hemoptysis has been defined as bleeding ranging from 100 to 1000 ml over a 24-hour period, although the range of 300–600 ml is more commonly recognized [2]. Accurately estimating the volume of hemoptysis is challenging, so the impact of respiratory distress or hypoxia on the patient is often more crucial. While there are various causes, the primary etiologies are bronchiectasis, necrotizing pneumonia, cancer, and tuberculosis (TB) [3, 4]. Massive hemoptysis is associated with a high mortality rate [5, 6], emphasizing the need for prompt diagnosis and treatment to prevent catastrophic consequences.

Since its introduction by Remy et al. in 1973 [7], bronchial artery embolization (BAE) has become a well-established, safe, and effective first-line therapy for severe hemoptysis [8]. As a minimally invasive procedure, it boasts a high technical and clinical success rate with minimal complications [9]. However, one disadvantage is the high recurrence rate [10]. Recently, bronchoscopic interventions have been widely applied in the management of massive hemoptysis, demonstrating a high success rate [11, 12]. The primary goal of bronchoscopic intervention treatment is to clear blood and secretions, maintain an unobstructed airway, prevent asphyxia, and achieve local hemostasis [13, 14]. Although bronchoscopy for massive hemoptysis carries the risk of exacerbating bleeding, it remains an effective diagnostic and therapeutic measure when necessary. Among bronchoscopic interventions, endobronchial tamponade with balloons or stents has been well-documented in case reports as an effective method for managing massive hemoptysis [15–17]. Currently, endobronchial tamponade is typically used as an interim therapy and removed within a short period, setting the stage for subsequent treatment [18]. BAE and endobronchial occlusion have both demonstrated effectiveness in managing massive hemoptysis. However, the detailed report on the efficacy of combination therapy has not been extensively studied. Therefore, the objective of this study is to investigate the safety and clinical efficacy of combined BAE and endobronchial tamponade therapy, providing valuable guidance for treatment selection.

## Methods

### Patient population

This study conducted a review analysis of patients with massive hemoptysis who were admitted to Fujian Medical University 2nd Affiliated Hospital between March 2018 and March 2022. The inclusion criteria were as follows: (1) age over 18 years old, (2) underwent BAE or combination therapy, and (3) had available follow-up data. The exclusion criteria included: (1) severe cardiac-cerebral vascular diseases (such as myocardial infarction, stroke etc.), (2) severe hepatorenal dysfunction (Child-Pugh C), (3) thrombocytopenia and/or coagulation disorders, (4) pulmonary embolism, (5) contraindications to BAE, such renal failure or uncontrollable respiratory failure and (6) women who were pregnant or lactating. A total of 67 patients were enrolled in the study. The enrolled patients were divided into two groups based on the therapy they received: the combination group and the BAE group. The study was approved by the Ethics Committee of Fujian Medical University 2nd Affiliated Hospital, and informed written consent was waived due to the retrospective nature of the study.

### BAE procedure

**Fig. 1:**
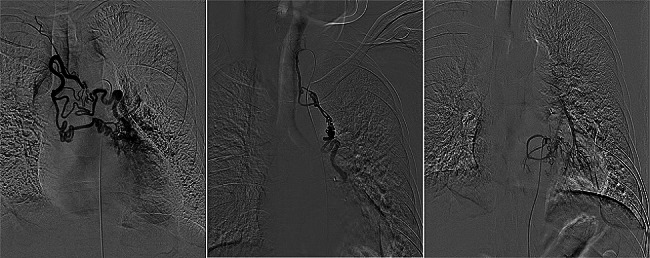
Angiographic image of a 51-year-old male patient with bronchiectasis

The procedure was performed using a 5-F vascular sheath inserted into the common femoral artery under local anesthesia. A pigtail catheter was then catheterized into the thoracic aorta using a modified Seldinger technique. A thoracic aortogram was conducted to confirm the condition of the bronchial arteries and identify any abnormal offender arteries. Selective angiography was performed after catheter insertion into the affected vessel (Fig. 1). Embolization was carried out when angiography revealed hypertrophic and tortuous arteries, neoangiogenesis, polyvascular supply, extravasation of contrast media, shunting into the pulmonary arteriovenous, tumor staining, or bronchial artery pseudoaneurysm. A microcatheter was introduced as distally as possible. Once the microcatheter was inserted into the targeted artery, embolic agents such as microspheres, microcoils, and/or gelfoam were injected.

The primary targets were technical and clinical success. Technical success was defined as the ability to catheterize and embolize the abnormal vessel. Clinical success was defined as the complete cessation of bleeding or a significant reduction in hemoptysis for at least 24 h, with no further intervention required for at least 1 month after BAE.

The BAE was performed in catheter room, pulmonologist with at least 10 years experiences performed BAE. The equipment includes: Cobra2 of 4–5 F, SIM1, TIG, multifunctional catheter, 2.4 F glassy microcatheter; Embolization equipment: Hengrui or Mairuitong 300–500 μm and 500–700 microspheres, gelatin sponge, and if necessary, combined with Boko’s spring coil.

### Endobronchial tamponade procedure

**Fig. 2:**
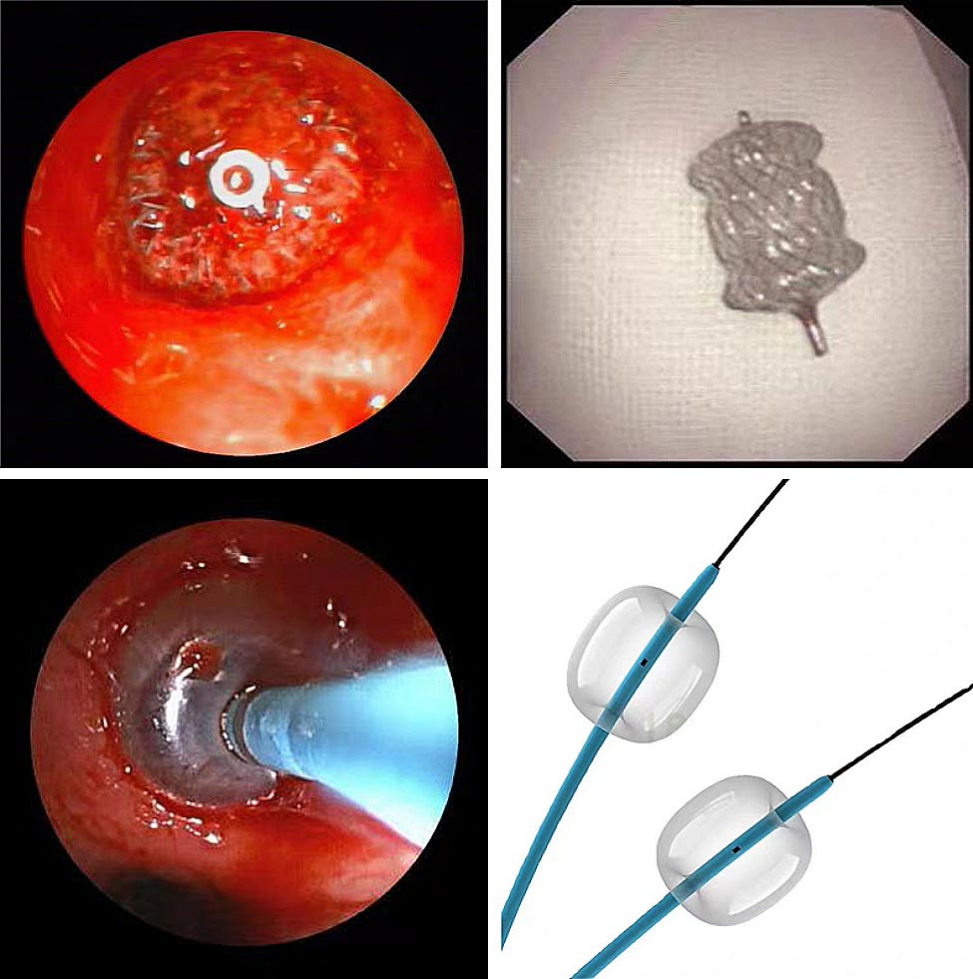
Endobronchial tamponade in patients with massive hemoptysis

The entire procedure was performed while monitoring the patient’s vital signs. Electronic bronchoscopy was conducted under local anesthesia, inserted through the nasal route. After removing secretions, blood, or blood clots and irrigating with cold saline solution, the site of continuous active bleeding was observed. Thrombin and epinephrine were locally applied at the bleeding site. A stent or balloon catheter was inserted through the operational channel of the bronchoscope and inflated inside the bronchus at the bleeding site to stop the bleeding (Fig. 2).

Procedure is performed by respiratory physicians who have worked for more than 10 years. The procedure is performed using intravenous sedation or general anesthesia, at the bedside of the emergency department or at a specialized respiratory intervention center. The diameter of the working channel selected for bronchoscopy should be above 2.8 mm. We first choose balloon filling, and if the balloon filling effect is not good, we choose stent occlusion.

### Data collection and follow-up

During the hospitalization period, data for outcome analysis were meticulously gathered from electronic medical records. The collected data encompassed a spectrum of crucial aspects for analysis, including patient demographic characteristics, etiology, comorbidities, procedural intricacies, adverse effects, alterations in blood gas analysis, and coagulation function indicators before and after treatment.

Following discharge, a structured regimen of regular telephone or clinical visit follow-ups was diligently adhered to, with the follow-up period extending until March 2023. Throughout these follow-ups, the recurrence and survival status of patients afflicted with hemoptysis were meticulously documented. Specifically, the date of any recurrence leading to readmission or mortality was meticulously recorded. Subsequently, calculations were performed to ascertain both hemoptysis-free survival and overall survival (OS) metrics. Hemoptysis-free survival was defined as the duration from the BAE procedure to the occurrence of hemoptysis recurrence, while OS denoted the interval from BAE to the eventual demise of the patient.

### Statistical analysis

Statistical software (SPSS 26.0) was used for data analysis. Categorical variables were compared using the χ2 test or Fisher’s exact test. The t-test or Wilcoxon test was employed for continuous variables. Both hemoptysis-free survival and overall survival were presented using the Kaplan-Meier curve. Cox regression analysis was conducted to identify the risk factors affecting hemoptysis-free survival and overall survival. A significance level of *P* < 0.05 was considered statistically significant.

## Results

### Basic characteristics

A total of 67 patients were included in this study, with 26 (38.8%) patients in the combination group and 41 (61.2%) patients in the BAE group. Table 1 summarizes the baseline characteristics of the patients. Bronchiectasis was the most common cause of massive hemoptysis in the study. There were no significant differences in age, sex, etiology, comorbidities, smoking history, culprit artery, non-bronchial artery involvement, and embolic agents between the combination group and the BAE group.

**Table 1:** Characteristics of study population

Characteristics	Combination group (*n* = 26)	BAE group (*n* = 41)	*P* value
Age (years), mean ± SD	61.50 ± 13.98	58.19 ± 13.46	0.338
Age group, No. (%)			0.183
≤ 60	9(34.6)	21(51.2)	
> 60	17(65.4)	20(48.8)	
Gender, No. (%)			0.977
Male	21(80.8)	33(80.5)	
Female	5(19.2)	8(19.5)	
Etiology, No. (%)			0.462
Bronchiectasis	14(53.8)	24(58.5)	
Necrotizing pneumonia	6(23.2)	9(22)	
Cancer	3(11.5)	7(17.1)	
Tuberculosis	3(11.5)	1(2.4)	
Comorbidities, No. (%)			
Hypertension	12(46.2)	17(41.5)	0.679
Diabetes	6(23.1)	5(12.2)	0.366
Chronic lung disease	13(50)	16(39)	0.377
Coronary heart disease	2(7.7)	2(4.9)	0.636
History of smoking, No. (%)			0.183
Yes	9(34.6)	21(51.2)	
No	17(65.4)	20(48.8)	
Number of arteries affected, No. (%)			0.317
1 vessel	10(38.5)	11(26.8)	
≥ 2 vessels	16(61.5)	30(73.2)	
Non-bronchial artery involvement			0.805
Yes	17(69.2)	28(70.7)	
No	9(30.8)	13(29.3)	
Embolic agents, No. (%)			
Microspheres	26(100)	41(100)	NA
Microcoils	11(42.3)	14(34.1)	0.501
Gelatin sponge	10(38.5)	12(29.5)	0.435
Hemoptysis (Mean)	261 ml	214 ml	0.154

BAE, bronchial artery embolization; SD, standard deviation

### Technical success and clinical success

The technical success rates for both the combination group and the BAE group were 100%. There was no significant difference in the clinical success rate between the combination group (92.3%) and the BAE group (87.8%) (*P* = 0.859). In terms of relapse rate, the combination group showed similar 3-month (3.8% vs. 4.9%, *P* = 0.842) and 6-month (11.5% vs. 19.5%, *P* = 0.603) hemoptysis relapse rates compared to the BAE group. However, a lower 1-year hemoptysis relapse rate was observed in the combination group (15.4% vs. 39.0%, *P* = 0.039) (Table 2). Furthermore, there were no significant differences between the groups in the 3-month (0% vs. 2.4%, *P* = 0.422), 6-month (3.8% vs. 4.9%, *P* = 0.841), and 1-year (7.7% vs. 14.6%, *P* = 0.393) hemoptysis mortality rates.

**Table 2:** Rates of success, relapse and death

Items	Combination group (*n* = 26)	BAE group (*n* = 41)	*P* value
Technical success, No. (%)	26(100)	41(100)	NA
Clinical success, No. (%)	24(92.3)	36(87.8)	0.859
Hemoptysis recurrence, No. (%)			
3-month	1(3.8)	2(4.9)	0.842
6-month	3(11.5)	8(19.5)	0.603
1-year	4(15.4)	16(39.0)	0.039
Mortality, No. (%)			
3-month	0(0)	1(2.4)	0.422
6-month	1(3.8)	2(4.9)	0.841
1-year	2(7.7)	6(14.6)	0.393

BAE, bronchial artery embolization

### Adverse events

None of the patients experienced serious adverse reactions. The adverse events in both the combination group and the BAE group were common, mild, and self-limited. Most patients had more than one adverse reaction. There were no significant differences in fever, chest discomfort, cough/expectoration, abdominal pain, and nausea/vomiting between the two groups (Table 3).

**Table 3:** Adverse effects

Items	Combination group (*n* = 26)	BAE group (*n* = 41)	*P* value
Fever	9(34.6)	8(19.5)	0.166
Chest discomfort	4(15.4)	11(26.8)	0.273
Cough/expectoration	10(38.5)	15(36.6)	0.877
Abdominal pain	0(0.0)	4(9.8)	0.266
Nausea/vomiting	0(0.0)	2(4.9)	0.518

BAE, bronchial artery embolization

### Indicators of blood gas analysis and coagulation function

There were no significant differences in PaO2, PaO2/FiO2, D-dimer (D-D), and fibrinogen (FIB) between the combination group and the BAE group before therapy (*P* > 0.05). On day 3 after treatment, PaO2, PaO2/FiO2, D-D, and FIB were significantly increased in all patients (*P* < 0.05). However, compared to the BAE group, all post-therapy indicators were higher in the combination group (*P* < 0.05) (Table 4).

**Table 4:** Indicators of blood gas analysis and coagulation function

Variables	Combination group (*n* = 26)	BAE group (*n* = 41)	*P* value
PaO_2_(mmHg), mean ± SD			
Before treatment	62.38 ± 16.45	65.50 ± 15.51	0.437
After treatment	88.94 ± 8.03	80.75 ± 10.75	0.001
P value	0.009	< 0.001	
PaO_2_/FiO_2_(mmHg), mean ± SD			
Before treatment	187.19 ± 35.61	200.80 ± 46.99	0.211
After treatment	306.19 ± 71.10	269.78 ± 56.69	0.027
P value	0.007	< 0.001	
D-dimer(mg/L), mean ± SD			
Before treatment	2.28 ± 2.70	3.32 ± 5.94	0.405
After treatment	6.32 ± 5.19	3.67 ± 4.11	0.023
P value	< 0.001	< 0.001	
Fibrinogen(g/L), mean ± SD			
Before treatment	3.23 ± 1.55	3.22 ± 1.34	0.982
After treatment	4.93 ± 1.56	4.05 ± 1.67	0.034
P value	0.007	0.031	

BAE, bronchial artery embolization; SD, standard deviation

### Hemoptysisfree survival and overall survival

**Fig. 3:**
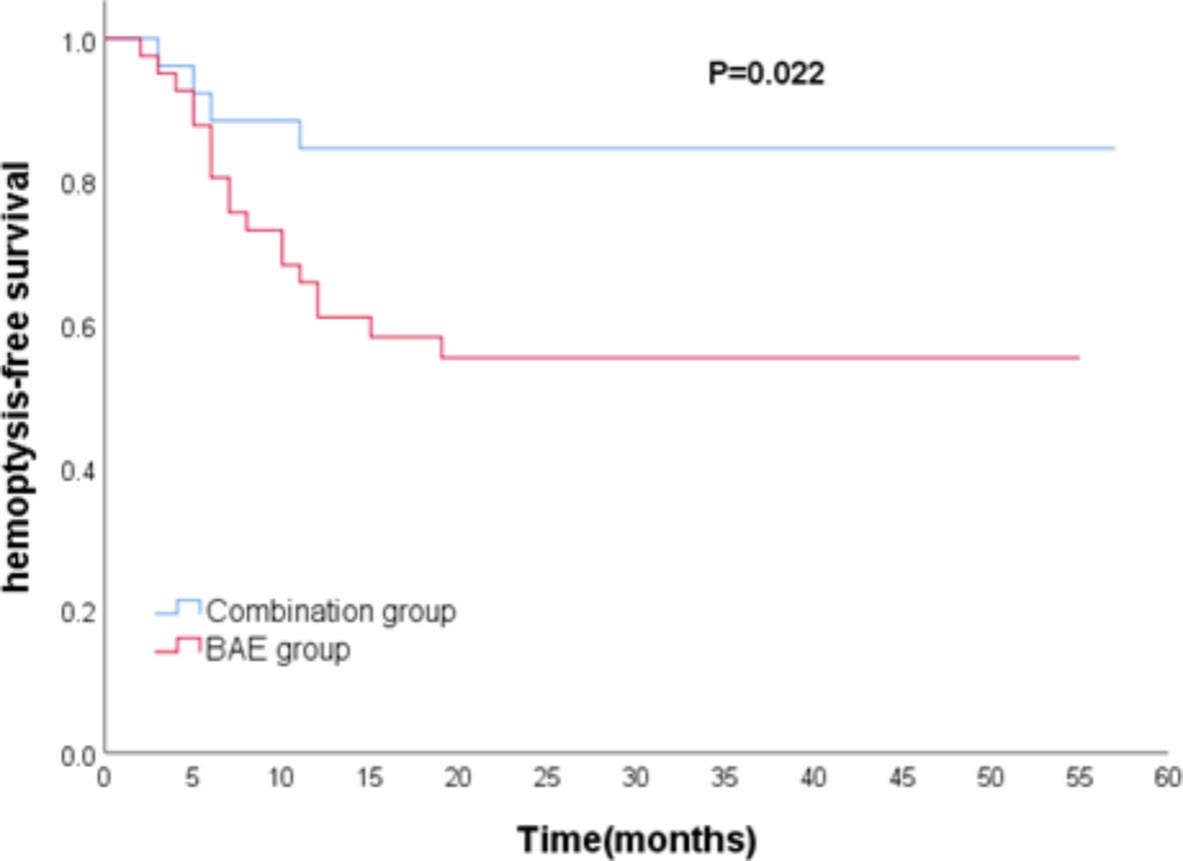
Comparison of hemoptysis-free survival between combination group and BAE group. BAE, bronchial artery embolization

**Fig. 4:**
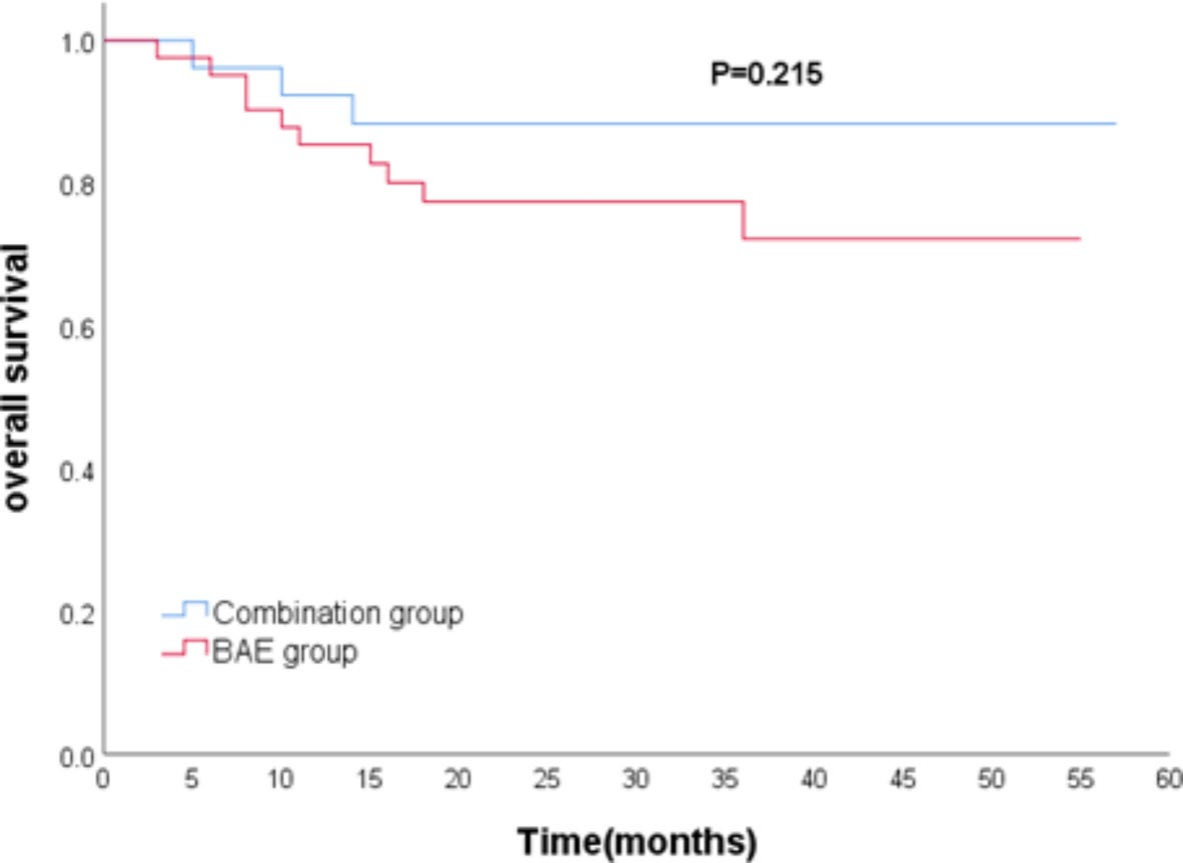
Comparison of overall survival between combination group and BAE group. BAE, bronchial artery embolization

The median follow-up time was 28 months as of the last follow-up in March 2023. Among the patients, 22 (32.8%) experienced hemoptysis recurrence (4 in the combination group and 18 in the BAE group), and 13 (19.4%) patients died (3 in the combination group and 10 in the BAE group). The median hemoptysis-free period was 28.5 months in the combination group and 18 months in the BAE group. The difference in hemoptysis-free survival between the two groups was statistically significant (*P* = 0.022, Fig. 3). The median overall survival in the combination group (30 months) did not differ significantly from the BAE group (24 months) (*P* = 0.215, Fig. 4).

**Fig. 5:**
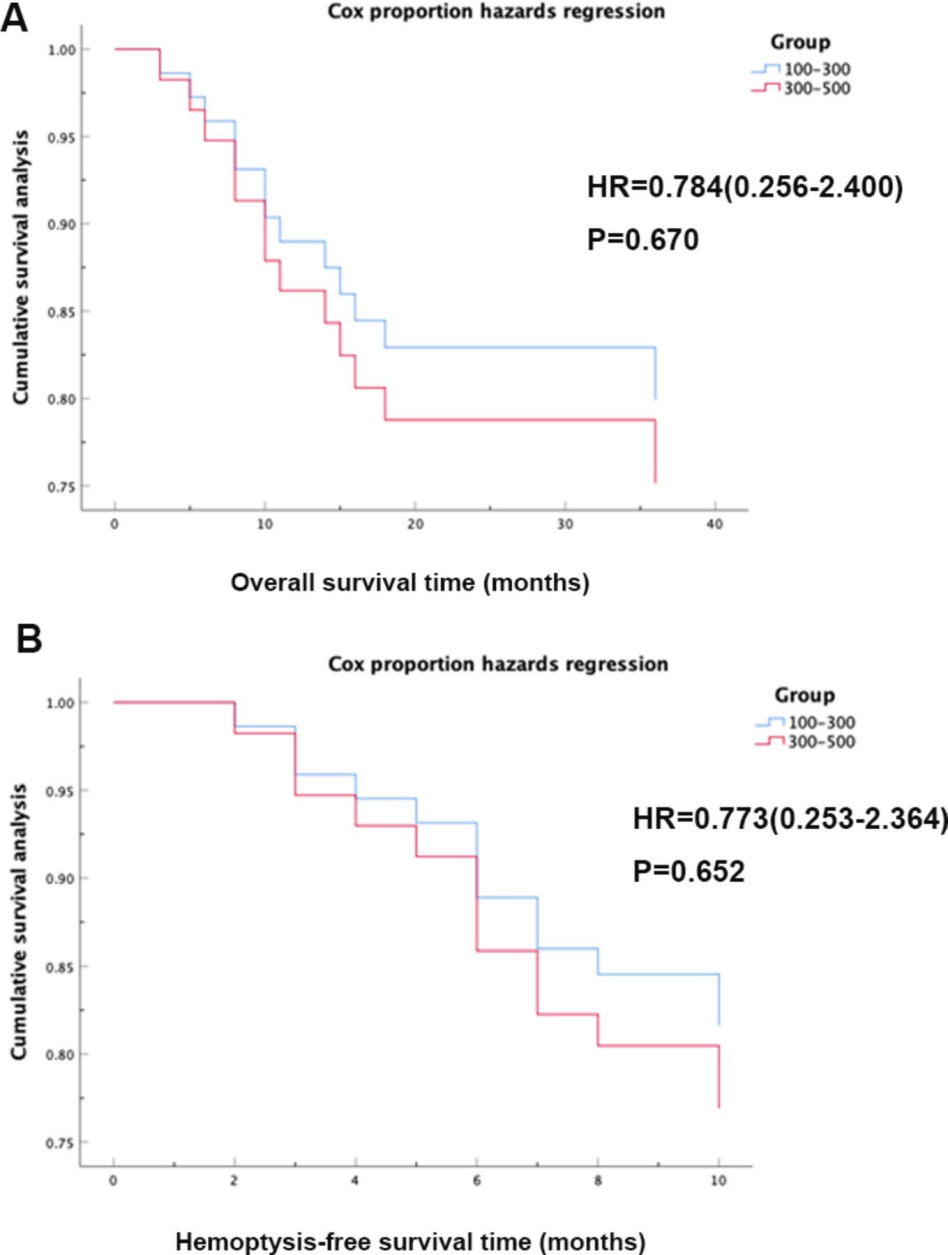
The Cox regression analysis. All patients were divided into two groups according to hemoptysis volume, 100-300 ml and 300-500ml. (**A**) The association between hemoptysis volume and overall survival (*P* = 0.670); (**B**) The association between hemoptysis volume and hemoptysis-free survival (*P* = 0.652)

Possible factors influencing hemoptysis-free survival are shown in Table 5. Univariable analysis indicated that combined therapy (HR 0.305, *P* = 0.032), etiology (HR 0.021, *P* < 0.001), number of affected arteries (HR 3.461, *P* = 0.046), and non-bronchial artery involvement (HR 5.742, *P* = 0.019) were significant factors. In the multivariable analyses, combined therapy (HR 0.314, *P* = 0.037), etiology (HR 0.015, *P* < 0.001), number of affected arteries (HR 3.677, *P* = 0.043), and non-bronchial artery involvement (HR 4.655, *P* = 0.047) remained significantly associated. Regarding factors influencing overall survival, both univariable and multivariable analysis indicated that only etiology (HR 0.039, *P* < 0.001) was significant (Table 6). The Cox regression analysis of showed that there was no significant difference between hemoptysis volume and overall survival (*P* = 0.670), and no significant difference between hemoptysis volume and hemoptysis-free survival (*P* = 0.652) (Fig. 5).

**Table 5:** Cox proportional hazards regression analysis of hemoptysis-free survival

Variables	Univariable			Multivariable		
	HR	95%CI	*P*	HR	95%CI	*P*
Combined therapy	0.305	0.103–0.903	0.032	0.314	0.106–0.931	0.037
Age	1.269	0.550–2.927	0.577			
Gender	0.836	0.283–2.471	0.746			
Etiology (others vs. malignant)	0.021	0.005–0.084	< 0.001	0.015	0.003–0.082	< 0.001
History of smoke	1.626	0.702–3.766	0.256			
Comorbidities	1.087	0.425–2.779	0.862			
Number of arteries affected	3.461	1.023–11.704	0.046	3.677	1.040-12.992	0.043
Non-bronchial artery involvement	5.742	1.341–24.593	0.019	4.655	1.018–21.282	0.047

HR, hazard ratio; CI, confidence interval; BAE, bronchial artery embolization

**Table 6:** Cox proportional hazards regression analysis of overall survival

Variables	Univariable			Multivariable		
	HR	95%CI	*P*	HR	95%CI	*P*
Combined therapy	0.452	0.124–1.643	0.228	0.691	0.183–2.616	0.587
Age	0.993	0.333–2.958	0.990			
Gender	1.089	0.299–3.973	0.897			
Etiology (others vs. malignant)	0.036	0.011–0.119	< 0.001	0.039	0.010–0.148	< 0.001
History of smoke	1.543	0.518–4.598	0.436			
Comorbidities	0.911	0.280–2.960	0.877			
Number of arteries affected	2.739	0.606–12.366	0.190	1.993	0.416–9.560	0.388
Non-bronchial artery involvement	2.586	0.573–11.680	0.217	0.805	0.144–4.491	0.805

HR, hazard ratio; CI, confidence interval; BAE, bronchial artery embolization

## Discussion

Endobronchial tamponade is a recognized technique for managing massive hemoptysis, offering advantages such as blood and secretion removal, airway patency, and prevention of asphyxia. It not only enables patients who were initially unable to undergo BAE to tolerate surgery but also ensures patient safety during transportation to interventional centers. Moreover, performing endobronchial tamponade before BAE allows for a longer treatment window for surgery.

BAE has emerged as a primary treatment option for critical, refractory, and recurrent hemoptysis [19]. It has shown favorable short-term results in the majority of cases [20]. Previous research has reported technical success rates ranging from 90 to 100% and immediate success rates ranging from 82 to 100% [21]. In this study, both patient groups demonstrated satisfactory success rates, with a technical success rate of 100% in both the combination group and the BAE group. It is possible that the small sample size contributed to the high success rate. Additionally, the immediate clinical success rates were similar between the two groups and fell within the reported range.

The recurrence of hemoptysis is a prevalent phenomenon post-BAE, given that BAE primarily offers palliative and symptomatic alleviation without tackling the root cause of the ailment [22, 23]. Recent studies have reported relapse rates ranging from 9.8 to 57.5% [24]. The timeframe for recurrence exhibits significant variability among individuals, ranging from as swiftly as 1 day to several years [25]. In our study, the documented recurrence incidence aligned with the previously reported spectrum. Notably, we observed a diminished 1-year recurrence rate in the combination group in contrast to the BAE group. Moreover, through multivariate analysis, we ascertained that combination therapy significantly curtailed the postoperative recurrence subsequent to BAE and elongated the duration of hemoptysis-free survival. Consistent with existing studies [26, 27], early recurrence predominantly stems from incomplete embolism of the pertinent arteries, whereas delayed recurrence is linked to the reconstruction of collateral circulation or the progression of the underlying pulmonary condition. Remarkably, the combination group exhibited a mortality rate comparable to that of the BAE group. The main cause of death after therapy was reported to be the potential progression of the disease [28]. It was further demonstrated in multivariate analysis that malignant disease was associated with mortality and OS.

Complications caused by bronchoscopy are uncommon [29]. The current rate of serious adverse events in BAE is relatively low [30]. This is attributed to experienced physicians, thorough preoperative examinations, and advanced equipment and materials [31]. In our study, none of the patients experienced serious adverse events. The adverse effects that did occur were relatively mild and well tolerated. The incidence of adverse effects in the BAE group was consistent with observations from previous research [32]. Additionally, no adverse effects related to bronchoscopy were observed in the combination group. Therefore, combined therapy appears to be a safe treatment option.

Patients with hemoptysis often succumb to suffocation and refractory hypoxemia caused by the presence of large amounts of blood in the alveoli, obstructing the airways [33]. PaO2 is a sensitive indicator of hypoxia, reflecting the external respiratory condition that determines the presence and severity of hypoxia [34]. Clinically, it reflects the actual ventilation function of lung tissues in a way and assess the degree of hypoxemia, which is used to assist in determining the clinical treatment effect [35, 36]. The results of this study indicated that patients in the combination group had higher PaO2 and PaO2/FiO2 values after treatment compared to the BAE group. This suggests that combination therapy significantly improved respiratory function and alleviated hypoxia.

D-dimer is a specific degradation product resulting from activation and hydrolysis of fibrin [37]. When thrombosis and fibrinolytic activity occur in the blood vessels of the body, it would increase significantly [38]. Fibrinogen (FIB) is a coagulation protein synthesized by the liver and is primarily involved in the blood clotting process [39]. Increased plasma fibrinogen level promotes platelet aggregation, which is conducive to thrombus formation [40]. BAE achieves the purpose of hemostasis by promoting thrombosis, which lead to increase D-dimer and FIB levels correspondingly [41]. This study showed that FIB and D-dimer levels were higher in the combination group than in BAE group after treatment. This suggests that combined therapy is more effective in achieving rapid hemostasis and symptom improvement, thus enhancing clinical efficacy.

Our study assessed the efficacy and safety of combination therapy for massive hemoptysis and further compared it with BAE, which had not been evaluated in previous studies. However, there were still some limitations in current study. First, since the study was retrospective, there may be biases in the data collection and analysis process, and factors influencing the effectiveness of combination treatments may have been overlooked. Second, the small sample size in this study may limit the statistical power and warrant the inclusion of more patients from multiple centers for further validation. Further studies with prospective design are necessary to validate the results and provide stronger evidence.

In conclusion, combination therapy appears to be more effective in improving respiratory function, correcting hypoxia, accelerating hemostasis, and reducing the recurrence rate of hemoptysis compared to embolization therapy alone. Therefore, combination therapy may be a preferable option for the treatment of massive hemoptysis and deserves wider clinical application.

## Data Availability

The data that support the findings of this study are available on request from the corresponding author. The data are not publicly available due to privacy or ethical restrictions.
